# Strain engineered pyrochlore at high pressure

**DOI:** 10.1038/s41598-017-02637-9

**Published:** 2017-05-22

**Authors:** Dylan R. Rittman, Katlyn M. Turner, Sulgiye Park, Antonio F. Fuentes, Changyong Park, Rodney C. Ewing, Wendy L. Mao

**Affiliations:** 1 Department of Geological Sciences, Stanford University, Stanford, California 94305 USA; 2 Cinvestav Unidad Saltillo, Apartado Postal 663, 25000 Saltillo, Coahuila Mexico; 3 High Pressure Collaborative Access Team, Geophysical Laboratory, Carnegie Institution of Washington, Argonne, IL 60439 USA; 4 Stanford Institute for Materials and Energy Sciences, SLAC National Accelerator Laboratory, Menlo Park, California, 94025 USA

## Abstract

Strain engineering is a promising method for next-generation materials processing techniques. Here, we use mechanical milling and annealing followed by compression in diamond anvil cell to tailor the intrinsic and extrinsic strain in pyrochlore, Dy_2_Ti_2_O_7_ and Dy_2_Zr_2_O_7_. Raman spectroscopy, X-ray pair distribution function analysis, and X-ray diffraction were used to characterize atomic order over short-, medium-, and long-range spatial scales, respectively, under ambient conditions. Raman spectroscopy and X-ray diffraction were further employed to interrogate the material *in situ* at high pressure. High-pressure behavior is found to depend on the species and concentration of defects in the sample at ambient conditions. Overall, we show that defects can be engineered to lower the phase transformation onset pressure by ~50% in the ordered pyrochlore Dy_2_Ti_2_O_7_, and lower the phase transformation completion pressure by ~20% in the disordered pyrochlore Dy_2_Zr_2_O_7_. These improvements are achieved without significantly sacrificing mechanical integrity, as characterized by bulk modulus.

## Introduction

Strain engineering is an effective method to manipulate the electronic structure of materials^1–5^, synthesize new materials^6, 7^, and enhance various properties of nano-scale materials^8, 9^. Multiple methods for introducing strain have been used, including mechanical milling^6, 7^, chemical doping^4^, substrate patterning^2^, monolayer buckling^1^, uniaxial tension^5^, biaxial compression^10^, and quasi-hyrdostatic compression^4, 11^. Some of these techniques (*e.g*., chemical doping and mechanical milling) are considered “intrinsic” since they increase strain without the continuous application of an external force. In contrast, other techniques (*e.g*., tension and compression) are “extrinsic” since the strain they apply is reliant on the continued application of said external force. Thus, intrinsic and extrinsic methods of strain engineering can be used in conjunction to further take advantage of the material modifications they induce.

The combination of mechanical milling, which has been shown to greatly affect the kinetics of phase transformations^6^, and quasi-hydrostatic compression by diamond anvil cell (DAC), which controls a fundamental thermodynamic parameter, pressure, provide two separate methods of controlling strain in materials. Mechanical milling followed by annealing provides a means to synthesize materials with varying amounts of intrinsic strain, while compression by DAC extrinsically increases strain in the material. Here, we have investigated the behavior of Dy_2_Ti_2_O_7_ and Dy_2_Zr_2_O_7_ pyrochlore oxides prepared by mechanical milling and subsequent annealing (to control defect concentration) under high-pressure conditions in a DAC. DACs allow materials to be studied *in situ* at high-pressure, providing the ability to gain a fundamental understanding of material behavior. This understanding can then lead to the optimization of other high-pressure processing techniques that can be applied to large volumes of material^12, 13^.

Pyrochlore (A_2_B_2_O_7_) complex oxides have shown utility in a wide range of technological applications. They have been proposed as nuclear waste forms for the immobilization of actinides^14^, electrolytes for solid oxide fuel cells^15, 16^, and quantum spin ices^17^. The behavior of defects in pyrochlore is important for many of these applications. As such, multiple methods for introducing defects into pyrochlore have been investigated in the past, including radiation damage^18–21^, high pressure^22–24^, and chemical doping^25–27^. A thorough understanding of the pyrochlore crystal structure is necessary to assess the behavior of defects in the material, and how those atomic-level defects can influence bulk properties. The intricate structure of pyrochlore oxides—with two cations that may or may not be ordered and coupling between the character of the cation and anion sub-lattices—also makes it an ideal candidate to study defects in from the perspective of fundamental science.

The ordered pyrochlore structure (*Fd-3m*) is a derivative of the AX_2_ fluorite structure (*Fm-3m*). Pyrochlore-structured materials have two distinct cation sites, as opposed to the ideal fluorite structure that has only one. Furthermore, 1/8 of the fluorite anion sites are vacant in the pyrochlore structure. These systematic vacancies are ordered, and they occupy a distinct site in the pyrochlore structure. The ordered pyrochlore structure forms when the ratio of the A and B cation radii is large (r_A_/r_B_ > 1.46). The A-site (*16d*) is eight-coordinated, while the B-site (*16c*) is six-coordinated, and the two cations alternate along the <110> direction. There are three distinct tetrahedrally coordinated anion sites: the *48f* site that is coordinated with two A and two B cations, the *8b* site that is coordinated with four A cations, and the vacant *8a* site that is coordinated with four B cations. The x-position of the *48f* site determines the shape of the A and B coordination polyhedra—an x-coordinate of 0.3125 indicates a perfect octahedral coordination for B, an x-position of 0.3750 indicates a perfect cubic coordination for A, and an intermediate value indicates some partial distortion of both polyhedra. These site labels are based on the origin being at the B-site^28, 29^.

The disordered pyrochlore structure, also known as defect-fluorite (*Fm-3m*), has one cation site and one anion site that is 7/8 occupied for pyrochlore compounds—the anion vacancy position is random. The defect-fluorite structure forms when the cationic radius ratio is small (r_A_/r_B_ < 1.46). The cation site is coordinated by eight anions, which are coordinated by four cations. The single cation site means that the lattice parameter of defect-fluorite is half that of pyrochlore, such that the pyrochlore structure is an ordered 2 × 2 × 2 superstructure of the disordered defect fluorite, which is evidenced by pyrochlore superstructure peaks in X-ray diffraction. Furthermore, the anion sub-lattice of the defect-fluorite structure is equivalent to the pyrochlore anion sub-lattice with a *48f* oxygen x-coordinate of 0.3750^28, 29^.

Subjecting pyrochlore to high pressure induces point defects—anti-site defects, where A and B cations switch sites, are the dominant type of cation defect, while Frenkel pairs are the dominant type of anion defect^30–32^. Although these defects play a critical role in the diverse applications of the pyrochlore structure-type^14, 33^, the influence of intrinsic defect concentration on the response of pyrochlore to extreme environments has received little attention^34, 35^.

Here, we investigate the effect of atomic structure, concentration of cation anti-site and anion Frenkel defects, and internal strain on the high-pressure structural response of Dy_2_Ti_2_O_7_ and Dy_2_Zr_2_O_7_. The amount of strain and defect concentrations were controlled by mechanical milling followed by annealing at various temperatures. The ordered pyrochlore, Dy_2_Ti_2_O_7_, with different initial defect concentrations, is compared with the inherently disordered pyrochlore, Dy_2_Zr_2_O_7_, which has the defect-fluorite structure. Atomic order in the samples at ambient conditions was characterized using X-ray pair distribution function (PDF) analysis, Raman spectroscopy, and X-ray diffraction (XRD). Raman spectroscopy and XRD were again used to probe the material *in situ* at high pressure. Results show that the increased intrinsic strain and defect concentrations can lower the onset pressure of the phase transformation in ordered pyrochlore, and lower the completion pressure of the phase transformation in disordered pyrochlore.

## Results

### Sample synthesis

Samples were annealed at high temperature following ball milling—an overview of all samples, along with shorthand notation for sample names to be used herein, is shown in Table 1. Annealing of anion defects occurs at a critical temperature of ~750 °C for titanate pyrochlore^36^ and ~1150 °C for zirconate pyrochlore^35^. Cations are increasingly ordered as the annealing temperature is increased^36^. For the ordered pyrochlore, Dy_2_Ti_2_O_7_, unannealed samples had small amounts of both cation and anion order, while samples annealed at 800 °C had increased cation order and large amounts of anion order. Samples annealed at 1200 °C had large amounts of both cation and anion order. Cation anti-site defect concentrations for DT12, DT8, and DT0 samples were 0.03(2), 0.15(4), and 0.24(5), respectively, as quantified by Rietveld refinement of XRD data. However, these values have many sources of error, including sensitivity of diffraction maxima intensity to the *48f* x-position, “spotty” diffraction from larger grains, and difficulty in defining a background due to the large amount of strain in DT8 and DT0. Furthermore, these anti-site defect concentrations deviate from previously reported values^36^. Even with these caveats, the qualitative trend of continuously lower anti-site defect concentrations at higher annealing temperatures is consistent with thermally-induced defect recovery. We note that the defect-fluorite Dy_2_Zr_2_O_7_ is inherently disordered due to its low radius ratio, but annealing temperatures of 1200 °C and 1500 °C were selected to be consistent with the behavior of ordered zirconate pyrochlore^35^.

**Table 1:** Sample descriptions and results summary.

Sample name	Composition	Annealing temperature [ °C]	Onset of transformation [GPa]	Completion of transformation [GPa]	Bulk modulus w/Bʹ = 8 [GPa]
DT12	Dy_2_Ti_2_O_7_	1200	34.2 (2.7)	not reached	259 (5)
DT8	Dy_2_Ti_2_O_7_	800	20.6 (1.8)	not reached	231 (5)
DT0	Dy_2_Ti_2_O_7_	N/A	ambiguous	not reached	178 (5)
DZ15	Dy_2_Zr_2_O_7_	1500	15.3 (1.3)	37.4 (1.2)	282 (15)
DZ12	Dy_2_Zr_2_O_7_	1200	15.2 (2.4)	not reached	282 (15)
DZ0	Dy_2_Zr_2_O_7_	N/A	17.1 (1.2)	29.9 (2.0)	282 (15)

Sample name, to be used as shorthand, indicates the composition of the sample as well as its annealing temperature. Summary of experimental results also provided.

### X-ray pair distribution function

**Figure 1:**
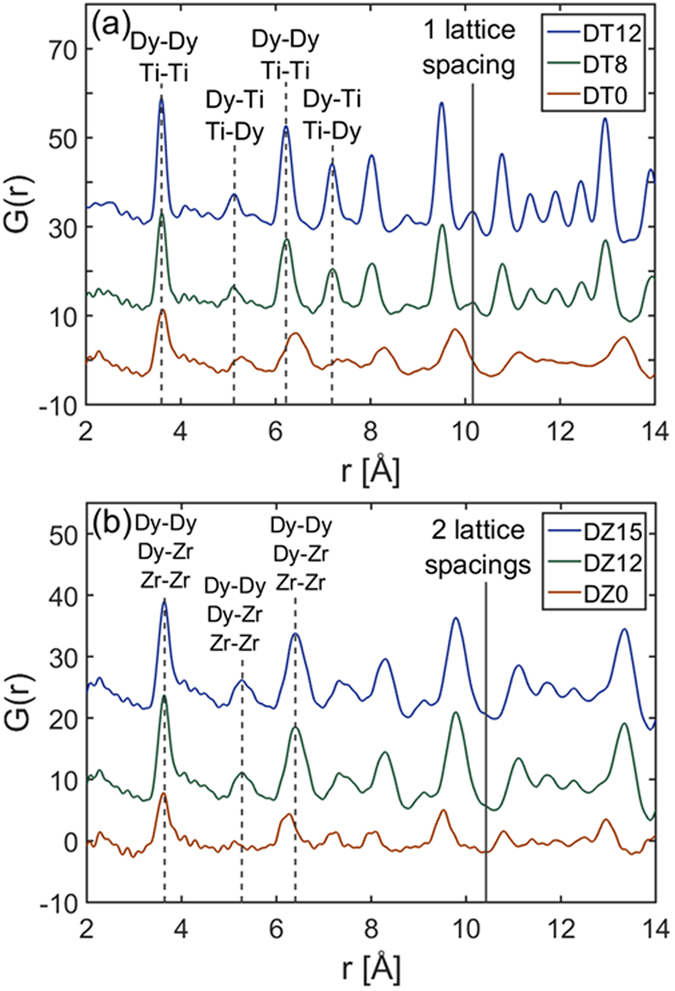
X-ray pair distribution functions of Dy_2_Ti_2_O_7_ and Dy_2_Zr_2_O_7_ at ambient pressure. X-ray pair distribution functions of (**a**) Dy_2_Ti_2_O_7_ and (**b**) Dy_2_Zr_2_O_7_ annealed at various temperatures. Data were recorded at ambient pressure. The lattice parameter and bond types are marked for both compositions. Ordering in Dy_2_Ti_2_O_7_ allows for differentiation in the types of bonds.

X-ray PDF analysis (Fig. 1) shows the effects of annealing on short-to-medium range order. Annealing of defects following mechanical milling causes ~2.9% unit cell contraction in Dy_2_Ti_2_O_7_, as can be observed by the systematic increase in bond length in DT0 as compared with DT8 and DT12 (Fig. 1a). This behavior is the opposite of that observed in Dy_2_Zr_2_O_7_, where annealing of defects leads to ~2.6% unit cell expansion (Fig. 1b). Lines used to mark bond distances in Fig. 1 are based on the peak centers of the samples annealed at the highest temperature. A breakdown in medium-range order is observed in DT0, with peaks at a distance past one unit cell showing substantial broadening. While this broadening is observed at a similar length-scale in DZ0, it is not as significant.

### X-ray diffraction

All samples undergo a high-pressure phase transformation to orthorhombic cotunnite (*Pnma*). Computational studies have shown that the cotunnite phase is a lower enthalpy structure than defect-fluortite or the compositionally equivalent amorphous phase for a variety of titanate and zirconate pyrochlore compositions at high pressure^37–40^. Experimentally, many single-cation fluorite-structured oxides have been shown to transform to cotunnite at high pressure^41, 42^. However, the large amounts of disorder and strain inherent to the high-pressure cotunnite structure of the two-cation pyrochlore makes it difficult to definitively identify through XRD^24, 43^. For consistency between datasets, onset of the phase transformation is defined by an increase in scattering intensity between the (222) and (004) pyrochlore structure diffraction maxima (equivalent to (111) and (002) in the defect-fluorite structure) because this is the location of the most intense cotunnite peaks^24, 43^. This is quantified in Supplementary Fig. S3, which shows the increase in X-ray scattering from cotunnite as a function of pressure.

**Figure 2:**
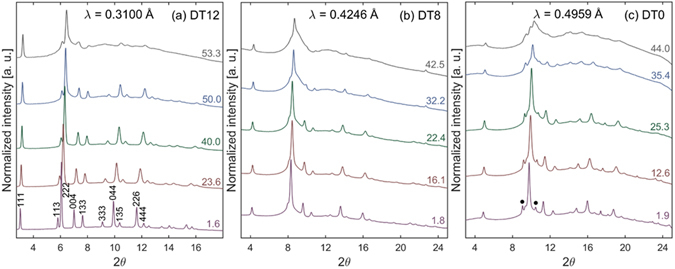
High-pressure X-ray diffraction of Dy_2_Ti_2_O_7_. Representative high-pressure XRD patterns for Dy_2_Ti_2_O_7_ annealed at various temperatures: (**a**) 1200 °C, (**b**) 800 °C, (**c**) unannealed. Miller indicies of the pyrochlore structure are shown in (**a**). A transformation from pyrochlore to cotunnite occurs at high pressure. Value on the right of each diffraction pattern is pressure in GPa. Black dots in (**c**) indicate the presence of excess Dy_2_O_3_ in DT0.

Selected XRD patterns from Dy_2_Ti_2_O_7_ at various pressures are shown in Fig. 2, with the complete dataset provided in Supplementary Fig. S1. DT8 and DT0 show evidence of increased strain at low pressures as compared with DT12—as indicated by the broad, diffuse background^36^—with DT0 showing more strain than DT8. All compounds experience a pressure-induced phase transformation. The onset of the phase transformation occurs at 34.2 GPa for DT12 and 20.6 GPa for DT8. An accurate transition pressure could not be identified in DT0 due to the large amount of strain even at low pressures. The increased scattering intensity between the (222) and (004) pyrochlore diffraction maxima at low pressure for DT8 and DT0 has been shown to originate from microstrain rather than an amorphous phase coexisting with pyrochlore^36^. The transformations are not complete for all Dy_2_Ti_2_O_7_ samples at the highest pressures studied.

**Figure 3:**
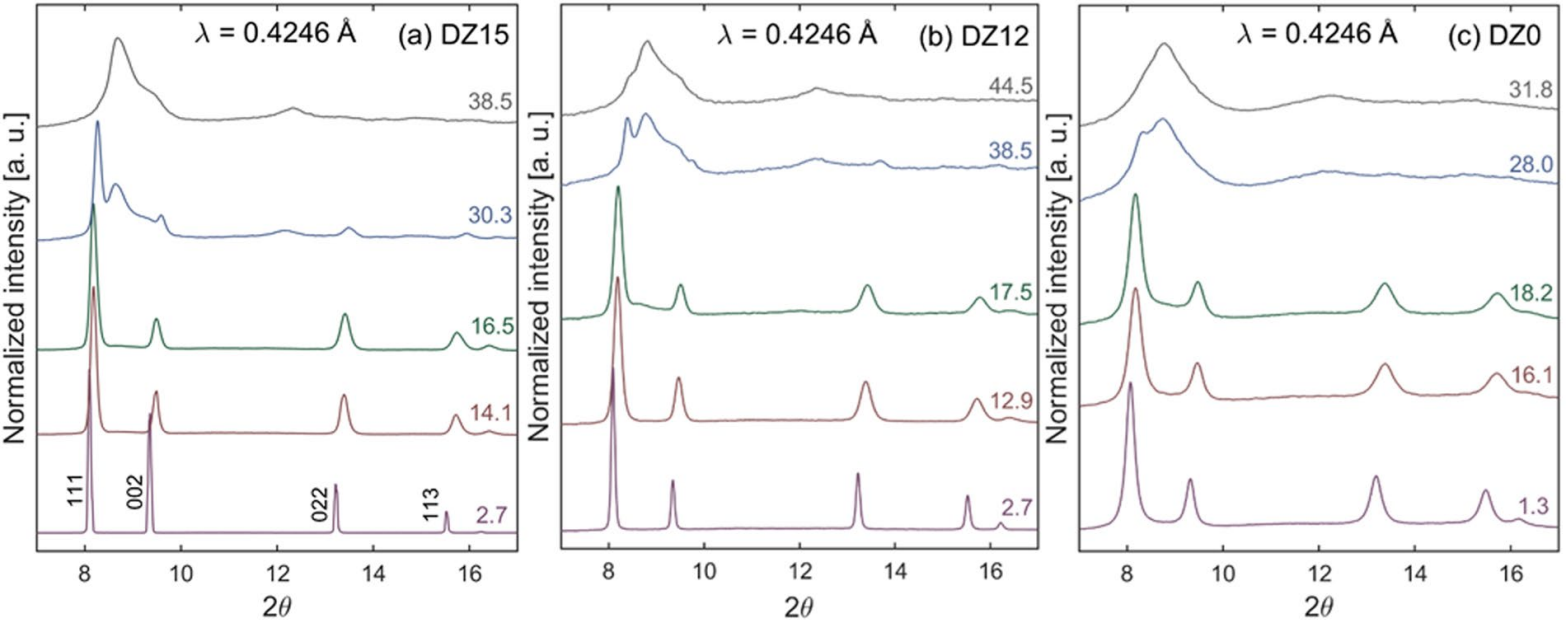
High-pressure X-ray diffraction of Dy_2_Zr_2_O_7_. Representative high-pressure XRD patterns for Dy_2_Zr_2_O_7_ annealed at various temperatures: (**a**) 1500 °C, (**b**) 1200 °C, (**c**) unannealed. Miller indicies of the defect-fluorite structure are shown in (**a**). A transformation from defect-fluorite to cotunnite occurs at high pressure. Value on the right of each diffraction pattern is pressure in GPa.

Selected XRD patterns from Dy_2_Zr_2_O_7_ at various pressures are shown in Fig. 3, with the complete dataset provided in Supplementary Fig. S2. DZ0 shows evidence of increased strain at low pressured as compared with DZ15 and DZ12—as indicated by broader peak width. However, the amount of strain in all Dy_2_Zr_2_O_7_ samples is less than what is seen in DT8 and DT0, as indicated by simple peak broadening as opposed to a broad, diffuse background. Similar to Dy_2_Ti_2_O_7_, all samples experienced a high-pressure phase transformation, likely to the cotunnite phase. The onset of the phase transformation is identified as 15.3, 15.2 and 17.1 GPa for DZ15, DZ12, and DZ0, respectively. Transformations are fully complete at 37.4 GPa for DZ15 and 29.9 GPa for DZ0. The transformation of DZ12 is not complete by 44.5 GPa, the highest pressure studied.

**Figure 4:**
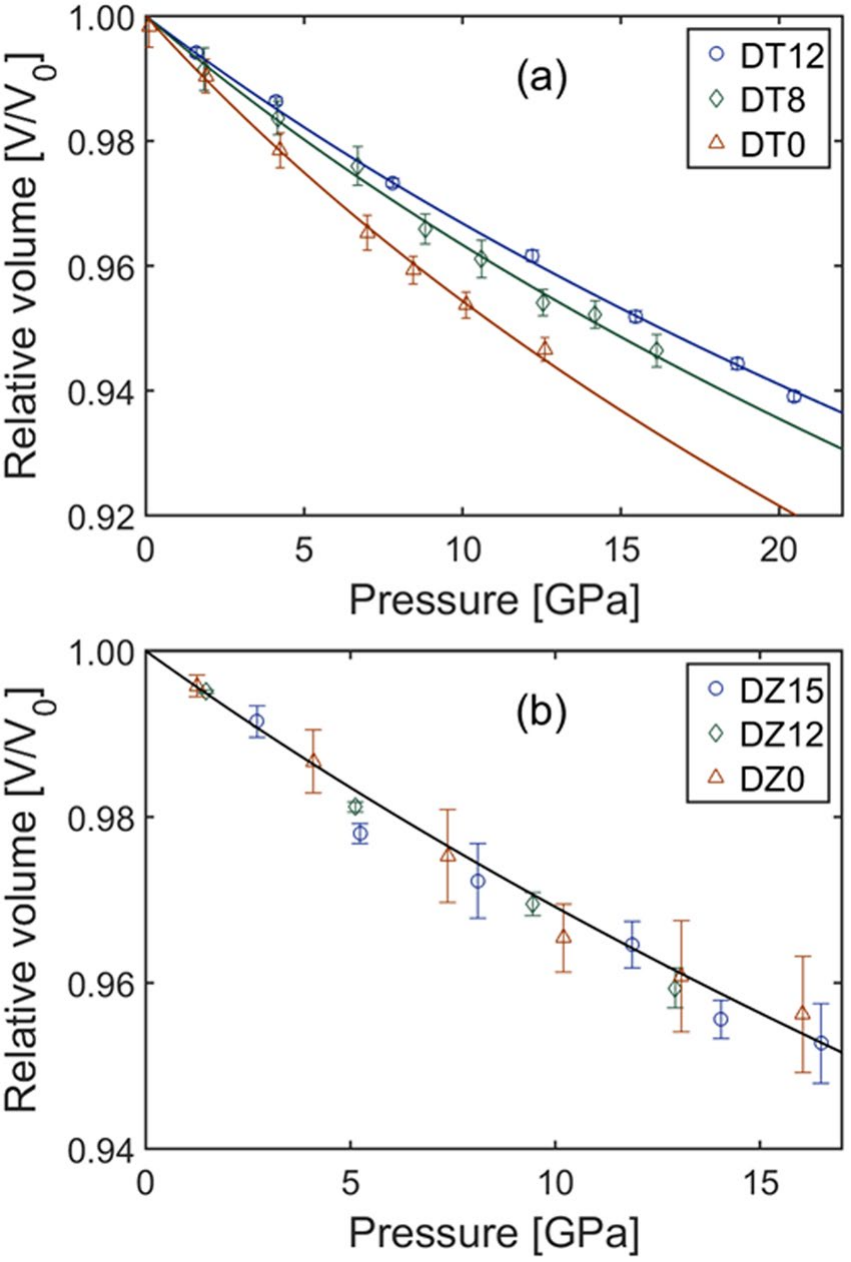
Compressibility curves of Dy_2_Ti_2_O_7_ and Dy_2_Zr_2_O_7_. Pressure-volume relationships for all (**a**) Dy_2_Ti_2_O_7_ and (**b**) Dy_2_Zr_2_O_7_ samples. Volumes are derived from Rietveld refinement of high-pressure X-ray diffraction data (Figs 2 and 3). Error in volume is due to positional variation in the diffraction maxima within a single diffraction pattern. Fittings to the pressure-volume data are performed using a second-order Birch-Murnaghan equation of state. Legends indicate the samples.

Pressure-volume curves for both Dy_2_Ti_2_O_7_ and Dy_2_Zr_2_O_7_ are plotted in Fig. 4a and 4b, respectively. Values of the plotted data are given in Supplementary Table S1. Data are plotted out to a maximum of ~15–20 GPa. This maximum pressure was chosen because plotting out to too high of a pressure can include effects from structural distortions due to the creation of the high-pressure cotunnite phase^44^, as well as increased non-hydrostaticity of the silicone oil pressure-transmitting medium^45^. Data are fit with a third-order Birch-Murnaghan equation of state using EosFit7-GUI^46, 47^ to determine the bulk moduli (B_0_) of the samples. The pressure-derivative of bulk modulus (Bʹ) is held constant at 8 for all samples so a direct comparison of B_0_ could be conducted. The bulk modulus of Dy_2_Ti_2_O_7_ is shown to increase with annealing temperature; B_0_ values for DT12, DT8, and DT0 were found to be 259(5), 231(5), and 178(5) GPa, respectively. In contrast, the bulk modulus of Dy_2_Zr_2_O_7_ was found to be within 282(15) for DZ15, DZ12, and DZ0. The large amount of scatter in the zirconate diffraction data makes it difficult to conclusively evaluate the bulk modulus of the individual samples, but plotted together a consistency between samples is clearly observed.

A summary of results—pressure of phase transformation onset/completion and bulk modulus—is given in Table 1.

### Raman spectroscopy

**Figure 5:**
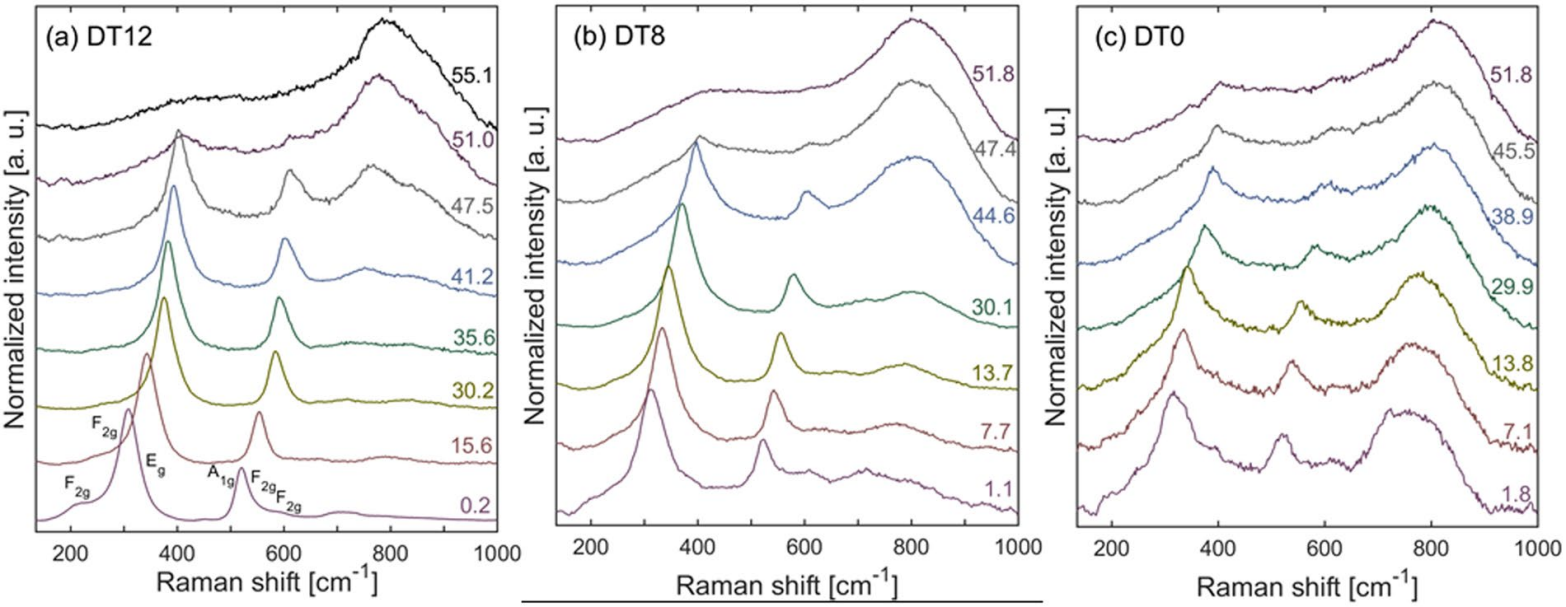
High-pressure Raman spectroscopy of Dy_2_Ti_2_O_7_. Representative high-pressure Raman spectra for Dy_2_Ti_2_O_7_ annealed at various temperatures: (**a**) 1200 °C, (**b**) 800 °C, (**c**) unannealed. Mulliken symbols of the Raman modes given in (**a**). Value on the right of each spectrum is pressure in GPa.

Raman spectra from Dy_2_Ti_2_O_7_ at various pressures are shown in Fig. 5. Raman modes prescribed to the pyrochlore structure are identified according to their Mulliken symbols in Fig. 5a ^48^. The broad mode between 700 and 800 cm^−1^ is not a fundamental Raman mode in pyrochlore-structured oxides, and is believed to be due to distortions to the BO_6_ octahedra^49, 50^. This mode is extremely weak in DT12 and extremely strong in DT0. The mode has moderate intensity in DT8, indicating that, even after heating past the critical temperature for anion defect annealing, there is still some increased distortion to the TiO_6_ octahedra. However, the amount of anion disorder in DT8 is much less than that of DT0.

**Figure 6:**
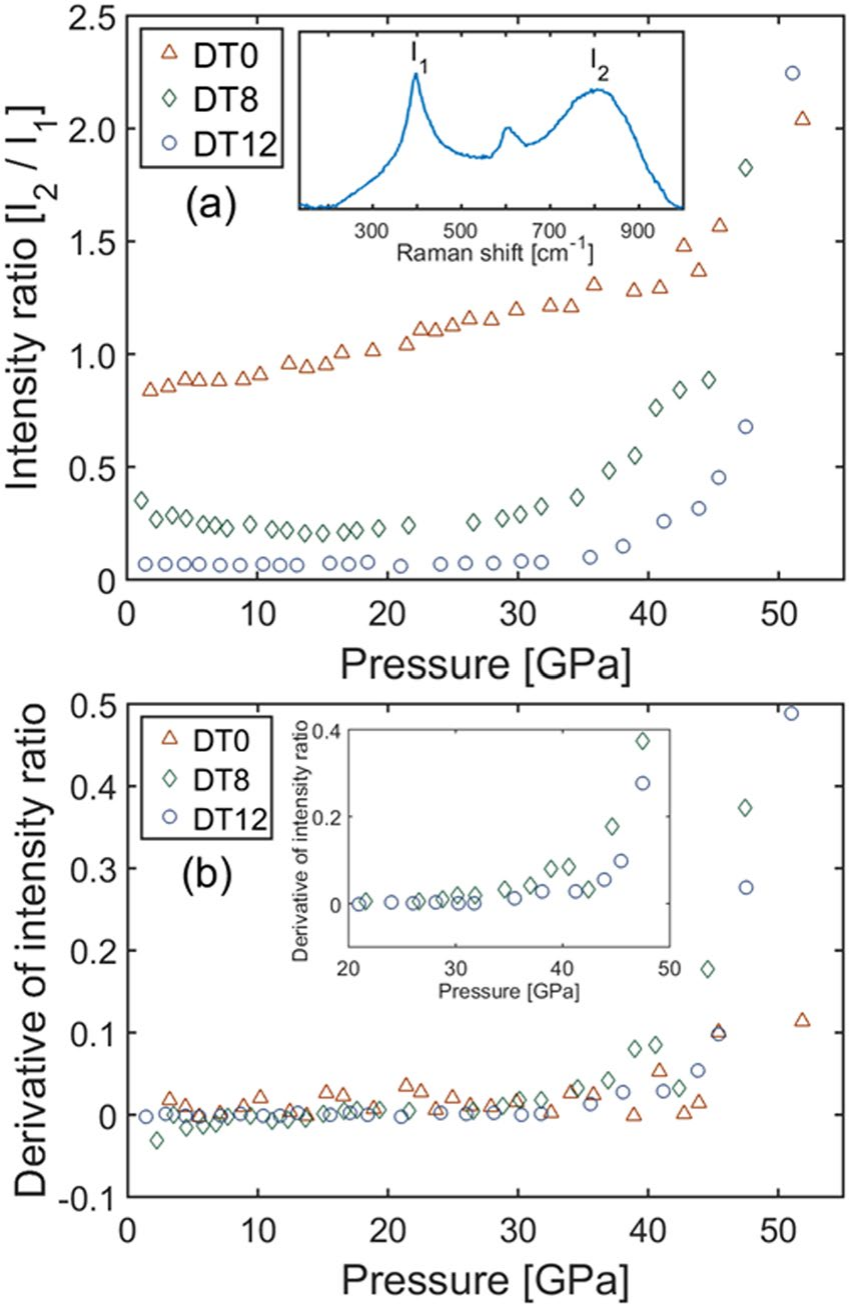
Pressure-induced anion disordering in Dy_2_Ti_2_O_7_. (**a**) Ratio of intensity between the Raman mode of the distorted TiO_6_ octahedra (I_2_) and the main F_2g_ Raman mode of the ordered pyrochlore (I_1_). This serves as a qualitative indicator of the progression of anion disorder as a function of pressure. Inset identifies the two modes in a representative high-pressure Raman spectrum. (**b**) Pressure derivatives of the intensity ratio data plotted in (**a**). This serves to better define the change in intensity ratio as a function of pressure. Inset focuses on the region of interest where the ratios begin to rapidly increase. Legends indicate the samples.

The ratio of intensity between the distorted TiO_6_ octahedra Raman mode and the main F_2g_ Raman mode at ω_0_ ≈ 309 cm^−1^ is shown in Fig. 6a. This is a qualitative measure of the amount of anion disorder in the sample as a function of pressure. The enhanced cation ordering and strain reduction in samples annealed at higher temperatures have little effect on the Raman spectra^36^. Thus, this ratio selectively indicates disorder on the anion sub-lattice.

A pressure derivative of these data, calculated using Heun’s method, are given in Fig. 6b. This provides a way to determine the pressure at which the intensity ratio begins to increase. The ratio for DT0 increases at a near constant rate, with a slight increase at the highest pressure measured. In contrast, the ratios of DT8 and DT12 are relatively constant in the low-pressure regime, followed by a rapid increase at higher pressures. DT8 experiences an initial increase at 30 GPa, with a more rapid increase beginning at 40 GPa. DT12 experiences an initial increase at 35 GPa, with a more rapid increase beginning at 44 GPa. Error in these values are approximately 2 GPa. Values of the data plotted in Fig. 6a and b are given in Supplementary Table S2.

Raman spectra of Dy_2_Zr_2_O_7_ could not be collected at high pressure due to the extremely weak signal even at long collection times. This may be related to the difficulties observed in collecting Raman spectra on powders of Dy_2_Hf_2_O_7_^51^, which is similar to Dy_2_Zr_2_O_7_ in its physical properties.

## Discussion

Marked differences in material properties, caused by differences in composition and annealing temperature, are observed at ambient conditions following synthesis under different conditions (Table 1). X-ray PDF data (Fig. 1) show that annealing of mechanically milled samples causes unit cell contraction of the Dy_2_Ti_2_O_7_ ordered pyrochlore structure, and unit cell expansion of the Dy_2_Zr_2_O_7_ disordered defect-fluorite structure. Contraction of the pyrochlore structure is expected since the mechanical milling process introduces anti-site defects. Anti-site defects have the larger A^3+^ cation replace the smaller B^4+^ cation in the octahedral polyhedron causing it to expand. Since this polyhedron acts as the framework of the pyrochlore structure^52^, decreased cation anti-site defects from annealing leads to unit cell contraction. Peak broadening and loss of medium-range order in DT0 is consistent with the large amount of defects in the structure, since increased anti-site defect concentration means there is increased variation in cation-cation bond distances due to a breakdown in the periodicity of alternating A^3+^ and B^4+^ cations along the <110> direction.

Expansion, as opposed to contraction, of the Dy_2_Zr_2_O_7_ unit cell following occurs because additional anti-site defects cannot be created since its structure is inherently disordered. Thus, oxygen atoms moving into interstitial sites (anti-Frenkel pairs) is the dominant defect type. Oxygen in their ideal sites act as a bridge between nearest-neighbor cations; that is, the two cations are bonded to the same oxygen. The movement of this oxygen to an interstitial site allows the cation-cation distance to contract, and annealing of the anti-Frenkel pair causes the cation-cation distance to expand back to its ideal value. The peak broadening and loss of medium-range order in DZ0 is less than that observed in DT0, consistent with the inability to further increase the anti-site defect concentration in defect-fluorite Dy_2_Zr_2_O_7_.

At moderate pressure—below the onset of the high-pressure phase transformation—material behavior is again seen to be dictated by its defects. Pressure-volume curves (Fig. 4) show that annealing temperature affects compressibility of Dy_2_Ti_2_O_7_, but not Dy_2_Zr_2_O_7_. This indicates that bulk modulus can be controlled by varying cation order—the trend of increasing bulk modulus with annealing temperature in Dy_2_Ti_2_O_7_ shows that cation ordering increases bulk modulus. This is again consistent with the description of the pyrochlore structure as a framework of interconnected BO_6_ octahedra. Distortions to these polyhedra work to decrease the material’s structural resistance to compression.

All samples transform to the cotunnite structure at high pressure. The commonly accepted mechanism for the pressure-induced pyrochlore-to-cotunnite phase transformation requires initial disordering of the anion sub-lattice before the transformation can proceed^50, 53, 54^. This anion disordering is tied to a decrease in the *48f* oxygen x-position, meaning that anion disorder can be thought of as the more octahedral coordination of the B cation in pyrochlore transitioning to the more cubic coordination environment typical of defect-fluorite. If the B-site is in fully cubic coordination, then the two cation sites are functionally equivalent in terms of their anion coordination environments, so the A and B cations no longer have a site preference. This facilitates the transformation to cotunnite, a phase with no cation site preferences^24, 43^. Consistent with this mechanism is that transition pressure increases with cationic radius ratio^23^ because a larger radius ratio increases the formation energy of both anion Frenkel pairs and cation anti-site defects^30–32^.

The data here are consistent with this mechanism for the transition. For instance, the Raman spectra of DT12 (Fig. 5a) shows that the anion sub-lattice is fully-disordered by 55.1 GPa, while there is still a large phase fraction of pyrochlore, as evidenced by the XRD pattern (Fig. 2a) at 53.6 GPa. And the onset of the high-pressure cotunnite phase in DT12 (34.2 GPa) corresponds well with the onset of anion disorder (35 GPa). Also, the transition pressure of DT12 is much higher than the transition pressure of DZ15, which is expected due to the larger cationic radius ratio of Dy_2_Ti_2_O_7_ as compared with Dy_2_Zr_2_O_7_^23^. However, this accepted transformation mechanism does not explain why DT12 has a higher transition pressure than DT8, or why DT8 begins to transform (20.6 GPa) before its anion sub-lattice begins to disorder (30 GPa). This can again be explained by examining the intrinsic defects in the sample at ambient conditions, and how these defects behave as pressure is increased.

In Dy_2_Ti_2_O_7_, the transition pressure of DT12 (34.2 GPa) is significantly higher than the transition pressure of DT8 (20.6 GPa); this nearly 50% decrease in transition pressure is accompanied by only a ~10% reduction in bulk modulus, showing that the energetics of the phase transformation can be improved without sacrificing significant mechanical integrity. The decrease in transition pressure is ascribed to increased disorder in DT8 relative to DT12, though this raises the question of whether it is the increased cation or anion disorder that lowers the transition pressure. The markedly different behavior of anion disordering in DT0 as compared with DT8 and DT12 in Fig. 6a suggests that DT8 and DT12 still have similar anion sub-lattice behavior despite the greater amount of anion disorder in DT8 at ambient conditions. This is consistent with the fact that anion defects are mainly annealed at a critical temperature, while cation defects are constantly annealed as temperature increases^36^. Thus, it is likely that increased cation disorder leading to strain lowers the transition pressure. In a well-ordered pyrochlore with little initial strain, disordering of anions is necessary in order to allow the phase transformation to occur. However, ample initial strain may sufficiently lower the enthalpy barrier of the phase transformation by increasing the internal energy of the pyrochlore phase such that anion disordering is no longer required^39^. In effect, controlling the defect concentration in pyrochlore at ambient conditions allowed for the decoupling of the anion and cation sublattices at high pressure, in addition to the decreased transition pressure.

In contrast to Dy_2_Ti_2_O_7_, the transition pressure of Dy_2_Zr_2_O_7_ is within error for all three samples—DZ15, DZ12, and DZ0. This consistency is due to the fact that the cation and anion sub-lattices of the defect-fluorite structure are inherently disordered under ambient conditions. However, increased strain is shown to improve the kinetics of the phase transformation, with the transition in DZ0 completed at a pressure 20% less than that of DZ15 (29.9 *vs*. 37.4 GPa). The sluggishness of the transformation in DZ12—incomplete by 44.5 GPa—relative to DZ15 is likely due to the much smaller grain size of DZ12^34^. Interestingly, this sluggishness does not extend to DZ0, which has an even smaller grain size than DZ12^55^, but completes its transformation at the lowest pressure. Peak broadening in Fig. 3 shows significantly higher amounts of strain on the cation sub-lattice in DZ0 as compared with DZ12 or DZ15. Thus, increased strain promotes the transition to the high-pressure phase to the point it overcomes the effect of the smaller grain size. This increase in kinetics is not accompanied by any reduction in bulk modulus, illustrating the preservation of the material’s mechanical integrity.

This mechanism of strain-enhanced phase transformation kinetics is consistent with swift heavy ion irradiation experiments where ZrO_2_ was irradiated *in situ* at high pressure^56^. ZrO_2_ undergoes a monoclinic-to-tetragonal phase transformation under irradiation, and a greater amount of tetragonal material was produced in samples irradiated at high pressure as compared with samples irradiated at ambient pressure. Furthermore, the amount of transformed material increased as pressure increased, substantiating the hypothesis that increased strain raises the enthalpy of the initial phase^57^, thus lowering the transition pressure. Both the monoclinic-to-tetragonal transformation in ZrO_2_ and the pyrochlore-to-cotunnite transformation in Dy_2_Ti_2_O_7_ are displacive^58, 59^, so there is a physical basis for the expectation that internal strain would help to drive these transformations.

We have demonstrated that the concentrations of defects in pyrochlore under ambient conditions—and, thus, the material’s intrinsic strain—influences its response to high pressure, an applied source of extrinsic strain. Increases in intrinsic strain are shown to lower the onset pressure of the phase transformation in the ordered pyrochlore Dy_2_Ti_2_O_7_, and lower the completion pressure of the phase transformation in the disordered pyrochlore Dy_2_Zr_2_O_7_. These changes in the material’s phase behavior are not associated with significant changes in mechanical integrity, as described by the bulk modulus of the material.

## Methods

### Sample synthesis

Dy_2_B_2_O_7_ (B = Ti, Zr) powders were prepared through solid-state synthesis^36^. Dy_2_O_3_ was mixed with BO_2_ in a 1:2 ratio and ball milled for 19 hours to create stoichiometric Dy_2_B_2_O_7_. The milled powders were then annealed in air for 12 hours at either 1200 °C or 800 °C for Dy_2_Ti_2_O_7_ and either 1500 °C or 1200 °C for Dy_2_Zr_2_O_7_. XRD confirmed that Dy_2_Ti_2_O_7_ crystallized as pyrochlore, and Dy_2_Zr_2_O_7_ crystallized as defect fluorite.

### High-pressure loading

Samples were compressed to high pressure (~40–50 GPa) using a symmetric DAC with 300 μm diameter diamond culets. Ruby fluorescence was used to monitor pressure^60^. Error in the ruby measurement is ~1 GPa above 40 GPa, but is otherwise less than 0.5 GPa. Stainless steel gaskets were pre-indented to 25 μm, and a 120 μm diameter hole was drilled to serve as a sample chamber. Silicone oil was used as the pressure-transmitting medium^45^.

### X-ray pair distribution function

X-ray PDF data were collected at beamline 11 ID-B of the Advanced Photon Source (APS), Argonne National Laboratory (ANL). X-ray energy was 90.50 keV (λ = 0.1370 Å). Polycrystalline powder samples were packed into polyimide capillaries (outer diameter of 0.0435” and length of 20 mm) sealed with epoxy. A large amorphous silicon-based area detector was used to record 2D diffraction images over a 120 s collection time. The raw diffraction images were integrated using the software FIT2d^61^ with a CeO_2_ standard for calibration. PDFgetX2^62^ was used to correct for background contributions, and to Fourier transform the data to generate the PDF, G(r).

### X-ray diffraction

Angular dispersive XRD data were collected at beamlines 16 BM-D of the APS, ANL, and 12.2.2 of the Advanced Light Source (ALS), Lawrence Berkeley National Laboratory (LBNL). X-ray energies used were 25 (λ = 0.4959 Å), 29.2 (λ = 0.4246 Å), and 40 keV (λ = 0.3100 Å). A CeO_2_ standard was used as a calibrant. Collection time was 30 or 60 s depending on signal intensity. A Mar345 image plate detector was used to record diffraction data. Integration of raw diffraction data was performed using Dioptas^63^. Maud was used to perform Rietveld refinement on the integrated diffraction patterns^64^. Representative Rietveld refinements for all samples are provided in Supplementary Fig. S4. Error in unit cell volume was quantified by fitting individual diffraction peaks in Fityk and taking the standard deviation of the volumes garnered from these peaks.

### Raman spectroscopy

Raman spectra were collected using a Renishaw RM1000 Raman microscope with 514.5 nm laser wavelength and 10 mW laser power. Data were summed over ten accumulations, each of which were 10 s in duration. Background subtraction was performed on the raw data before normalization. This background was attributed to scattering from the diamonds since it was largely absent from spectra recorded at ambient conditions.

Heun’s method, used to calculate the pressure-derivative of the intensity ratio (Fig. 6b), is defined as:

$$\frac{dIR}{dP}=\frac{1}{2}(\frac{I{R}_{i+1}-I{R}_{i}}{{P}_{i+1}-{P}_{i}}+\frac{I{R}_{i}-I{R}_{i-1}}{{P}_{i}-{P}_{i-1}})$$

where *IR* is intensity ratio, P is pressure, *i* is the index of the data point, and *dIR/dP* is the change in intensity ratio with respect to pressure. This equation was modified to calculate *dIR/dP* at the endpoints, where either *i* + *1* or *i* − *1* does not exist, by only calculating the slope in one direction.

## Electronic supplementary material

Rittman 2017 Supplementary Information
