# Supplementary material for: Strain engineered pyrochlore at high pressure
Source: Sci Rep. 2017 May 22;7:2236. doi: 10.1038/s41598-017-02637-9 (PMC5440404; doi:10.1038/s41598-017-02637-9)
Supplement: Supplementary file 1 — Rittman 2017 Supplementary Information [file 41598_2017_2637_MOESM1_ESM.pdf]

Strain engineered pyrochlore at high pressure

Dylan R. Rittman[1,*], Katlyn M. Turner[1], Sulgiye Park[1], Antonio F. Fuentes[2], Changyong Park[3], Rodney C. Ewing[1], Wendy L. Mao[1,4]

[1] Department of Geological Sciences, Stanford University, Stanford, California 94305, U.S.A.

[2] Cinvestav Unidad Saltillo, Apartado Postal 663, 25000 Saltillo, Coahuila, Mexico

[3] High Pressure Collaborative Access Team, Geophysical Laboratory, Carnegie Institution of Washington, Argonne, IL 60439, U.S.A.

[4] Stanford Institute for Materials and Energy Sciences, SLAC National Accelerator Laboratory, Menlo Park, California 94025, U.S.A.

* Corresponding author: (Dylan R. Rittman) Electronic mail: drittman@stanford.edu

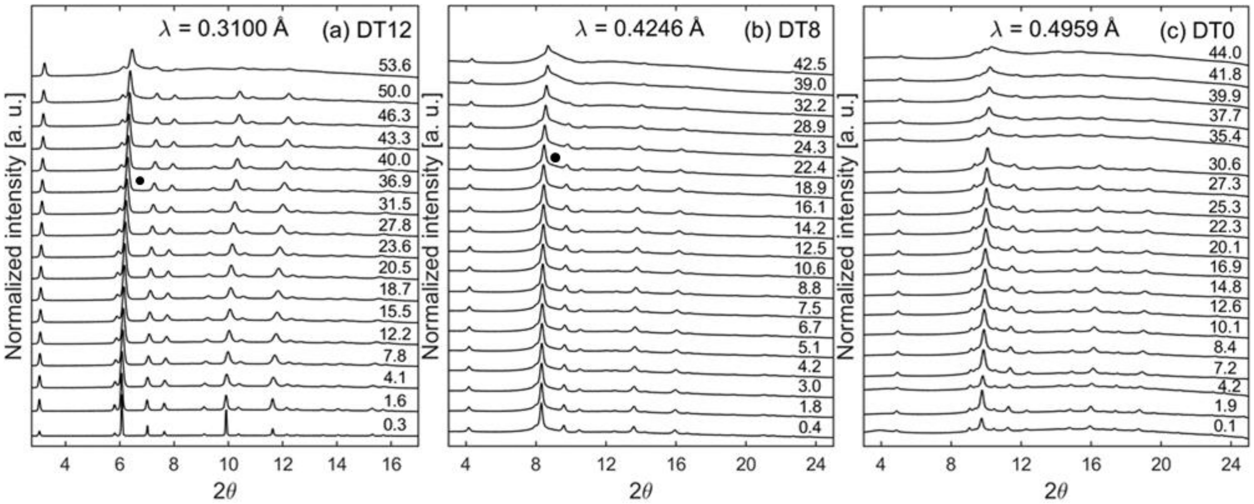

Supplementary Fig. S1. Complete set of $Dy_2Ti_2O_7$ X-ray diffraction data. This is provided in addition to the selected X-ray diffraction patterns shown in Fig. 2. Black dots indicate the onset of the transformation.

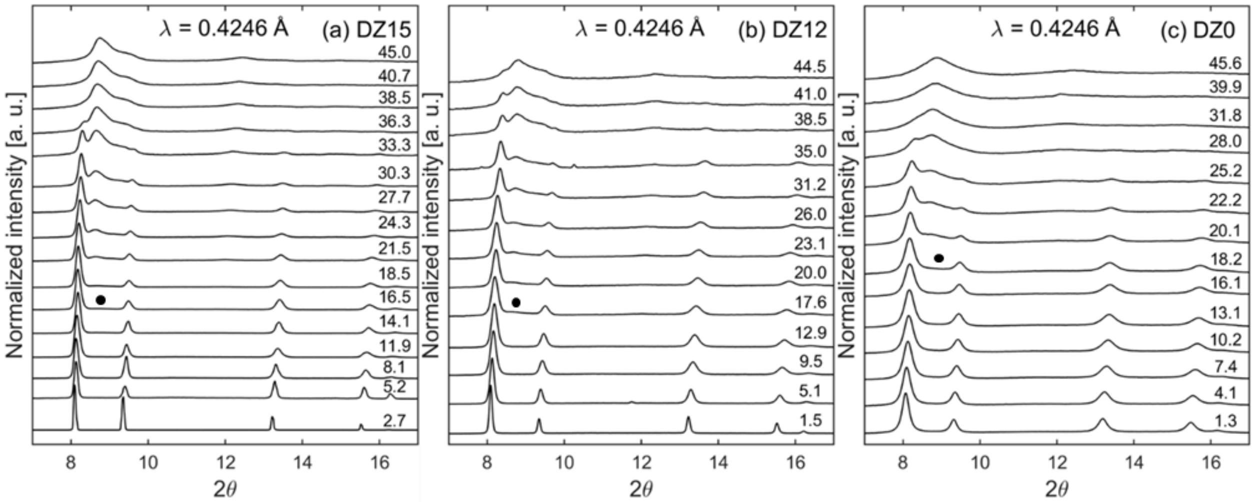

Supplementary Fig. S2. Complete set of $Dy_2Zr_2O_7$ X-ray diffraction data. This is provided in addition to the selected X-ray diffraction patterns shown in Fig. 3. Top: full two-theta range of XRD data. Black dots indicate the onset of the transformation.

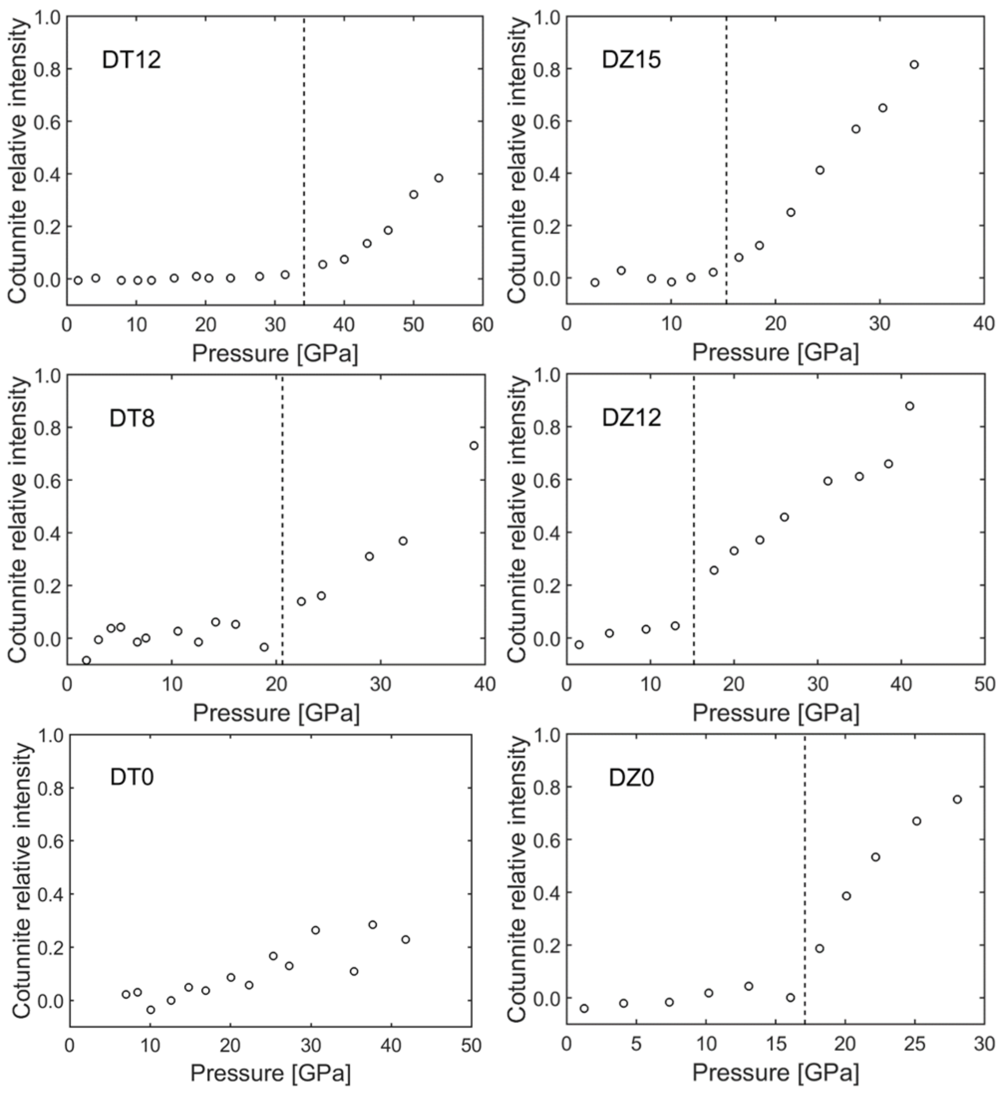

Supplementary Fig. S3. Quantitative cotunnite phase fraction as a function of pressure. These data provide a quantitative basis for the onset pressures of the phase transformation quoted in Table I. Pyrochlore intensity is defined as the total peak area in the (222) and (004) diffraction maxima. Cotunnite intensity is defined as the total peak area between the (222) and (004) pyrochlore diffraction maxima, after microstrain (based on low-pressure diffraction) has been subtracted. We note that these values may not represent the absolute phase fraction of cotunnite in the sample; but they reflect that an increase in cotunnite phase fraction begins to occur after onset pressures of the phase transformation quoted in Table I.

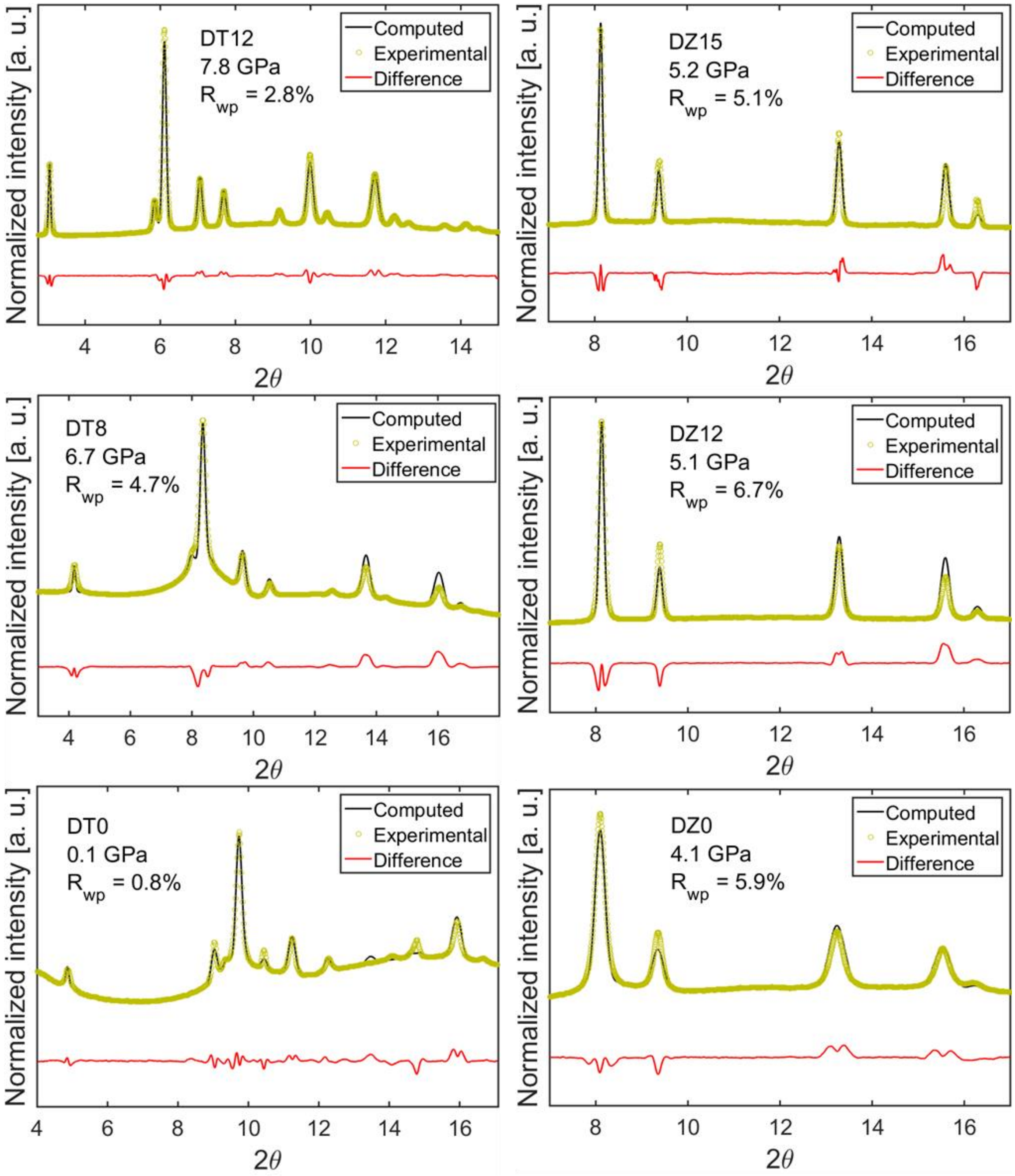

Supplementary Fig. S4. Representative Rietveld refinements for each sample. Rietveld refinement was used to calculate the unit cell volumes of the pyrochlore and defect-fluorite structures, which were determined through the refinement of the unit cell parameter. Deviations from theoretical diffraction intensities do not affect this value.

Supplementary Table SI. Data from Fig. 4. Numerical values of the pressure-volume data plotted in Fig. 4.

DT12			DT8			DT0		
Pressure [GPa]	Volume [Å^3]	Error [Å^3]	Pressure [GPa]	Volume [Å^3]	Error [Å^3]	Pressure [GPa]	Volume [Å^3]	Error [Å^3]
1.6	1044.10	0.72	1.8	1045.08	3.60	0.1	1046.07	3.46
4.1	1035.94	0.61	4.2	1036.83	2.70	1.9	1037.79	2.82
7.8	1022.23	0.74	6.7	1028.69	3.27	4.2	1025.31	2.90
12.2	1009.88	0.89	8.8	1018.03	2.51	7.0	1011.46	2.94
15.5	999.75	0.91	10.6	1012.99	3.18	8.4	1005.21	2.30
18.7	991.68	0.99	12.5	1005.60	2.18	10.1	999.38	2.24
20.5	986.23	0.79	14.2	1003.60	2.28	12.6	991.87	2.04
			16.1	997.51	2.78			

DZ15			DZ12			DZ0		
Pressure [GPa]	Volume [Å^3]	Error [Å^3]	Pressure [GPa]	Volume [Å^3]	Error [Å^3]	Pressure [GPa]	Volume [Å^3]	Error [Å^3]
2.7	141.49	0.27	1.5	141.67	0.03	1.3	143.00	0.19
5.2	139.56	0.17	5.1	139.69	0.08	4.1	141.69	0.55
8.1	138.75	0.65	9.5	138.02	0.20	7.4	140.06	0.80
11.9	137.65	0.41	12.9	136.60	0.34	10.2	138.63	0.59
14.1	136.36	0.33	17.6	135.41	0.63	13.1	137.97	0.96
16.5	135.96	0.69	20.0	134.01	0.69	16.1	137.32	1.01
18.5	135.52	0.76				18.2	137.02	0.86
21.5	134.79	0.78				20.1	135.94	1.11

Supplementary Table SII. Data from Fig. 6. Numerical values of the Raman intensity ratio data and its pressure derivative data plotted in Fig. 6.

DT12			DT8			DT0		
**Pressure [GPa]**	**Intensity Ratio**	**Derivative**	**Pressure [GPa]**	**Intensity Ratio**	**Derivative**	**Pressure [GPa]**	**Intensity Ratio**	**Derivative**
0.2	0.0759	-0.0075	1.1	0.3518	-0.0755	1.8	0.8385	0.0123
1.4	0.0670	-0.0028	2.2	0.2672	-0.0314	3.2	0.8560	0.0182
3.0	0.0699	0.0010	3.5	0.2832	0.0001	4.4	0.8860	0.0091
4.4	0.0701	-0.0011	4.5	0.2704	-0.0154	5.5	0.8797	-0.0019
5.5	0.0675	-0.0018	5.8	0.2464	-0.0127	7.1	0.8828	0.0014
7.2	0.0653	-0.0008	6.8	0.2400	-0.0102	8.9	0.8844	0.0098
8.7	0.0649	0.0011	7.7	0.2273	-0.0022	10.2	0.9082	0.0199
10.4	0.0691	-0.0012	9.5	0.2432	-0.0016	12.5	0.9556	0.0033
11.7	0.0630	-0.0013	11.1	0.2225	-0.0076	13.8	0.9366	-0.0014
13.1	0.0658	0.0023	12.3	0.2193	-0.0060	15.3	0.9540	0.0264
15.6	0.0721	-0.0002	13.7	0.2065	-0.0052	16.5	1.0049	0.0227
17.7	0.0677	0.0018	15.0	0.2049	0.0014	18.9	1.0145	0.0069
18.5	0.0771	-0.0002	16.6	0.2113	0.0052	21.4	1.0395	0.0350
21.0	0.0596	-0.0018	17.6	0.2177	0.0056	22.5	1.1069	0.0275
24.0	0.0696	0.0022	19.3	0.2257	0.0058	23.7	1.1011	0.0057
26.0	0.0717	0.0010	21.6	0.2416	0.0046	25.0	1.1226	0.0205
28.1	0.0736	0.0023	26.6	0.2528	0.0051	26.3	1.1555	0.0102
30.2	0.0814	-0.0001	28.8	0.2704	0.0108	27.9	1.1492	0.0092
31.8	0.0754	0.0014	30.1	0.2885	0.0179	29.9	1.1926	0.0151
35.6	0.1014	0.0127	31.8	0.3248	0.0179	32.5	1.2132	0.0024
38.1	0.1484	0.0270	34.6	0.3627	0.0320	34.1	1.2085	0.0265
41.2	0.2578	0.0284	37.0	0.4841	0.0423	35.8	1.3058	0.0237
43.8	0.3149	0.0541	38.9	0.5520	0.0802	38.9	1.2794	-0.0010
45.4	0.4529	0.0978	40.6	0.7602	0.0851	40.9	1.2925	0.0530
47.5	0.6780	0.2766	42.4	0.8406	0.0319	42.7	1.4767	0.0009
51.0	2.2472	0.4886	44.6	0.8841	0.1775	43.9	1.3652	0.0146
			47.4	1.8258	0.3736	45.5	1.5645	0.1007
						51.8	2.0387	0.1138
